# Conducting and Magnetic Hybrid Polypyrrole/Nickel Composites and Their Application in Magnetorheology

**DOI:** 10.3390/ma17010151

**Published:** 2023-12-27

**Authors:** Marek Jurča, Jarmila Vilčáková, Natalia E. Kazantseva, Andrei Munteanu, Lenka Munteanu, Michal Sedlačík, Jaroslav Stejskal, Miroslava Trchová, Jan Prokeš

**Affiliations:** 1 University Institute, Tomas Bata University in Zlín, 760 01 Zlín, Czech Republic; jurca@utb.cz (M.J.); vilcakova@utb.cz (J.V.); kazantseva@utb.cz (N.E.K.); munteanu@utb.cz (A.M.); strouhalova@utb.cz (L.M.); msedlacik@utb.cz (M.S.); 2 University of Chemistry and Technology, 166 28 Prague, Czech Republic; miroslava.trchova@vscht.cz; 3 Faculty of Mathematics and Physics, Charles University, 180 00 Prague, Czech Republic; jprokes@semi.mff.cuni.cz

**Keywords:** nickel microparticles, polypyrrole, hybrid composites, resistivity, conductivity, magnetization, magnetorheology

## Abstract

Hybrid organic/inorganic conducting and magnetic composites of core–shell type have been prepared by in-situ coating of nickel microparticles with polypyrrole. Three series of syntheses have been made. In the first, pyrrole was oxidised with ammonium peroxydisulfate in water in the presence of various amounts of nickel and the composites contained up to 83 wt% of this metal. The second series used 0.1 M sulfuric acid as a reaction medium. Finally, the composites with polypyrrole nanotubes were prepared in water in the presence of structure-guiding methyl orange dye. The nanotubes have always been accompanied by the globular morphology. FTIR and Raman spectroscopies confirmed the formation of polypyrrole. The resistivity of composite powders of the order of tens to hundreds Ω cm was monitored as a function of pressure up to 10 MPa. The resistivity of composites slightly increased with increasing content of nickel. This apparent paradox is explained by the coating of nickel particles with polypyrrole, which prevents their contact and subsequent generation of metallic conducting pathways. Electrical properties were practically independent of the way of composite preparation or nickel content and were controlled by the polypyrrole phase. On the contrary, magnetic properties were determined exclusively by nickel content. The composites were used as a solid phase to prepare a magnetorheological fluid. The test showed better performance when compared with a different nickel system reported earlier.

## 1. Introduction

Hybrid organic/inorganic composites constitute the core of many functional materials. This applies especially to the cases when both matrix/filler components have a functional character, i.e., they display complementary chemical or physical properties alone or in accord. The combination of metals with conducting polymers in composites is one of the possibilities frequently discussed in the literature in the design of energy conversion and storage devices and in a variety of other applications. Even though the composites applied in practice often include additional inorganic components, typically mixed metal oxides and sulfides or carbons [1,2], the understanding of binary systems is essential. One of the open research tasks includes the preparation of polypyrrole/nickel composites.

Nickel is a key element used in electrodes for its conductivity and electrocatalytic activity, suitable morphology and material properties. Furthermore, its magnetic properties make the element even more attractive. Conducting polymers, such as polypyrrole, have often been used as additives that improve conductivity and electrochemical activity and facilitate electron–ion transfers [2]. The system, which is composed of metallic nickel and a conducting polymer, polypyrrole, is discussed below.

Nickel metal may be present in two forms: nickel foam and microparticles, the former being dominant. Nickel foam has been used as a support for the deposition of polypyrrole by the in-situ chemical oxidation of pyrrole with ammonium peroxydisulfate [3,4,5,6]. An original approach was represented by the chemical oxidation using silver nitrate at nickel foam that catalysed the pyrrole polymerisation and introduced metallic silver at the same time [7]. The polypyrrole coating was obtained in an acidic solution even without an added oxidant [8]. In another case, the separately prepared polypyrrole suspension was simply deposited in nickel foam [9]. As an alternative, the nickel foam has been coated with polypyrrole potentiostatically [10,11], at a constant current density [12] or by using cyclic voltammetry [13,14]. Systems based on nickel foam are applicable, especially as current collectors in supercapacitors where the nickel provides the conductivity as well as the mechanical support for the deposition of polypyrrole [4,5,6,7,8,9,15,16,17]. Other diverse applications are represented by electrodes for electrocatalytic oxidation of methanol [11], improved water-splitting [14,17], solar steam-generation [3,10], solar-thermal desalination [18], or allowed for controlled electrosorption of organic pollutant dye [12].

There are two types of composites based on polypyrrole and nickel microparticles. The first is represented by the deposition of nickel on conducting polymer. Polypyrrole was electrochemically prepared on a graphite electrode, followed by the electrochemical electrodeposition of nickel microparticles [19,20,21]. This type of polypyrrole/nickel particulate composite has been used as an electrocatalyst of hydrogen-evolution reaction [17,19] or ethanol oxidation [20], and in the design of glucose biosensors [21] or in insulin sensors [22]. The reverse strategy relies on the coating of nickel particles with polypyrrole. Polypyrrole was electrodeposited on nickel particles at silicon nanowire arrays [16]. The chemical oxidation of pyrrole with sodium peroxydisulfate in the presence of nickel flakes is another example [23].

The defined preparation of polypyrrole-coated nickel microparticles is still a challenge. Virtually any interface immersed in the aqueous reaction mixture used for the preparation of conducting polymers by the chemical oxidation of respective monomers becomes coated with a thin submicrometre polymer film. This applies both to polyaniline and polypyrrole. The oligomers produced in the early stages of oxidation are hydrophobic, and they adsorb at available surfaces. The growth of polymer chains follows, resulting in the brush-like coating of substrates with conducting polymers. Such an approach has also been used for the coating of inorganic particles, e.g., ferrites [24]. When applied to particles of non-noble metals, such as nickel, some problems have been met. The oxidation of pyrrole to polypyrrole proceeds invariably under acidic conditions. The combination of an acidic medium and the presence of an oxidant is expected to cause the corrosion of non-noble metals followed by their dissolution, instead of being coated with a conducting polymer. For this reason, studies in this direction have been rare [23,25]. For example, when aniline hydrochloride was oxidised with ammonium peroxydisulfate in the presence of nickel microparticles, polyaniline/nickel was produced at reduced yield and conductivity of the order 10^−2^–10^−3^ S cm^−1^, i.e., values below the conductivity of components [25]. This phenomenon was explained by the nickel dissolution assisted by sulfuric acid generated during polymerisation. It was accompanied by the evolution of hydrogen gas catalysed by nickel and followed by a simultaneous reduction of the emeraldine form of polyaniline to leucoemeraldine. The present study reports similar trends in the preparation of polypyrrole/nickel composites but offers an alternative explanation of their properties.

Polypyrrole/nickel composites, where nickel core is coated with polypyrrole shell, may find use in various electrodes, suspensions in magnetorheology, magnetically separable adsorbents, electrocatalysts, fillers in functional flexible composites, and electromagnetic interference shielding, thus exploiting also magnetic and catalytic properties of nickel [26]. The preparation of such composites and their electrical characterisation is the goal of the present study, which introduces a novel way to include the deposition of polypyrrole nanotubes.

## 2. Experimental

### 2.1. Preparation

Nickel microparticles with a hedgehog-like morphology were employed as a conducting and magnetic filler to prepare polypyrrole-coated nickel microparticles (Table 1). Nickel particles (99.8%; Goodfellow, London, UK) exhibited a normal size distribution with a mean diameter of 5.5 ± 1.8 μm. Various amounts of nickel were dispersed in an aqueous solution of pyrrole, and then the solution of ammonium peroxydisulfate, also in water, was added under stirring to initiate the in-situ polymerisation of pyrrole. The concentrations of reactants were 0.1 M pyrrole (1.34 g, 20 mmol per 200 mL) and 0.125 M ammonium peroxydisulfate (5.71 g, 25 mmol per 200 mL). The polymerisation was left to proceed for 30 min at room temperature. In the second series, water was replaced with 0.1 M sulfuric acid (Table 1). The resulting composite microparticles of globular polypyrrole deposited on nickel were separated by filtration, rinsed several times with distilled water or 0.1 M sulfuric acid, followed by ethanol, to remove any unreacted reagents or by-products. The solids were left to dry at ambient temperature in open air for 48 h. The preparation of similar composites, including polypyrrole nanotubes and nickel, followed the same protocol in water that contained, in addition, 0.004 M methyl orange (262 mg, 0.8 mmol per 200 mL). All chemicals were supplied by the Sigma-Aldrich branch (Prague, Czech Republic) and used as delivered.

### 2.2. Composition

The composition of prepared materials involved the combustion of the organic component in an oxygen atmosphere at 800 °C in a muffle furnace (Nabertherm L9/S27, Lilienthal, Germany). The residual mass represented by nickel(II) oxide was recalculated to nickel metal. Additionally, a subset of randomly selected samples was subjected to thermogravimetric analysis (TA Q500; TA Instruments, Eden Prairie, MN, USA) with a heating rate of 10 °C min^−1^ in an oxygen atmosphere. The results were consistent with those obtained by the bulk combustion method.

### 2.3. Morphology

A scanning electron microscope (Nova NanoSEM FEI, Brno, Czech Republic) was used to assess the morphology of polypyrrole and its composites with nickel. Prior to analysis, the samples were gold sputter-coated using a JEOL JFC 1300 Auto Fine Coater (JEOL, Tokyo, Japan).

### 2.4. Spectroscopy

ATR FTIR spectra were analysed with a Nicolet 6700 spectrometer (Thermo-Nicolet, Waltham, MA, USA) equipped with a reflective ATR extension GladiATR (PIKE Technologies, Fitchburg, WI, USA) and a diamond crystal. Spectra were recorded in the range of 4000–400 cm^−1^ with a resolution of 4 cm^−1^, 64 scans, and Happ–Genzel appodization. The OMNIC 8 package was used for both the spectrometer control and spectral data processing.

Dispersive Raman spectra were recorded in a back-scattering geometry using a Scientific DXR Raman microscope (Thermo Fisher Scientific, Waltham, MA, USA) using a 780 nm excitation laser line. The scattered light was analysed by a spectrograph with holographic grating 1200 lines mm^−1^ and a 50 μm pinhole width. The acquisition time was 10 s with 10 repetitions.

### 2.5. Resistivity

A four-point van der Pauw method using a lab-made press based on a cylindrical glass cell with an inner diameter of 10 mm was used to assess electrical properties [27]. The powdered composites were placed between a support and a glass piston with four wire electrodes fixed at its perimeter. The experimental setup included a current source, a Keithley 220, a Keithley 2010 multimeter and a Keithley 705 scanner with a Keithley 7052 matrix card (Keithley Instruments Inc., Cleveland, OH, USA). The pressure up to 10 MPa (=102 kp cm^−2^) was registered with a L6E3 strain gauge cell (Zemic Europe BV, Etten-Leur, The Netherlands). The pressure was applied with an E87H4-B05 stepper motor (Haydon Switch & Instrument Inc., Waterbury, CT, USA). The sample thickness was recorded during the compression with a dial indicator Mitutoyo ID-S112X (Mitutoyo Corp., Sakado, Japan). The resistivity was also separately determined on composite pellets prepared after compression at 527 MPa by a manual hydraulic press (Specac, Orpington, UK).

### 2.6. Magnetic Properties

The magnetic characteristics were determined by measurement of a magnetic hysteresis curve in the range ±10 kOe by a vibrating sample magnetometer (VSM, Model 7407, Westerville, OH, USA).

### 2.7. Magnetorheology

The fluids were prepared by mixing the polypyrrole/nickel composites with mineral oil (Sigma-Aldrich, Czech Republic; viscosity 19.6 mPa s at 25 °C) at 9 wt% composite content. After preparation and before each measurement, the suspensions were sonicated in an ultrasonic bath for 10 min to remove any agglomerates. For the rheological measurements, the rotational rheometer Physica MCR 502 (Anton Paar, Graz, Austria) was used. To determine the flow properties of the samples under an external magnetic field, the device was equipped with an MRD 170/1T magneto cell and H-PTD hood. Magnetic fields varied from 0 to 1050 kA m^−1^. For the tests, a 20 mm plate–plate geometry with a sandblasted surface was used to reduce the potential wall slip. The measurements were performed at a 300 μm gap and 25 °C, which was controlled using an external bath. Two types of flow tests were performed. At first, the flow curves were obtained for various magnetic fields. The shear rate window used was determined to be between 0.01 and 150 s^−1^ to avoid overflow. The second type of measurement included a stepwise increase of the magnetic field under steady shear. The magnetic field was turned on and off periodically every 20 s while the magnetic field linearly increased by 150 kA m^−1^ with the shear rate kept constant at 50 s^−1^. Before each measurement, the samples were redispersed under the shear rate of 50 s^−1^ for 1 min, and after each test, the magnetic field was set to 150 kA m^−1^ to avoid potential sedimentation. Each measurement was repeated at least twice.

## 3. Results and Discussion

### 3.1. Preparation

**Figure 1:**
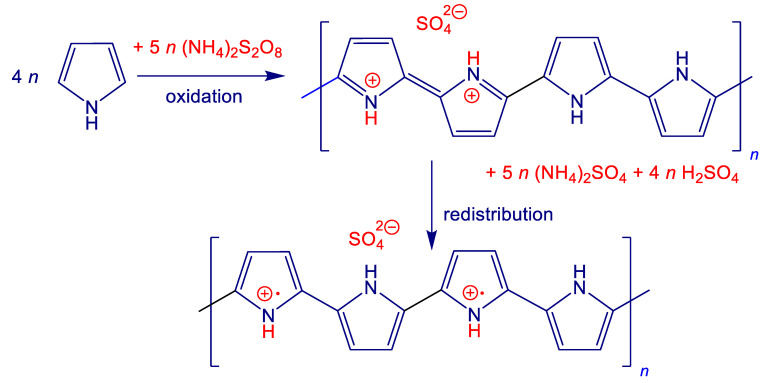
The oxidation of pyrrole with ammonium peroxydisulfate yields polypyrrole (sulfate salt). The electron redistribution generates the polaronic structure. Ammonium sulfate and sulfuric acid are by-products.

It should be noted that polymer chemists prefer the use of iron(III) chloride as an oxidant of pyrrole due to the superior conductivity of polypyrrole, especially in the preparation of polypyrrole nanotubes. When pyrrole was oxidised with iron(III) chloride in the presence of nickel microparticles, however, nickel dissolved and no composite was obtained. In an attempt to reduce the acidity of the reaction medium, the oxidant was replaced by ammonium peroxydisulfate. There was a single report in the literature when polypyrrole coating of nickel flakes was achieved by admicellar polymerisation of pyrrole, i.e., by the oxidation of pyrrole with sodium peroxydisulfate in the presence of anionic surfactant micelles [23]. The conductivity of coated nickel has not changed, and nickel has not dissolved either. When used in a polyethylene matrix, improved particle contacts afforded by polypyrrole coating were found. Following this approach, pyrrole was oxidised with ammonium peroxydisulfate in aqueous medium along with various portions of nickel microparticles and conducting polypyrrole/nickel composites have been obtained in stoichiometric yield (Figure 1). In contrast to the case of polyaniline [25], no hydrogen evolution associated with a partial metal dissolution of nickel has been observed.

The oxidation of pyrrole produces polypyrrole salt (Figure 1). The constitutional repeating unit is represented by four pyrroles with two protonated nitrogen atoms. The redistribution of electrons yields cation radicals (polarons) within the chain structure. They act as charge carriers that are responsible for the electrical conduction. When ammonium peroxydisulfate is an oxidant, sulfuric acid and ammonium sulfate are by-products. A part of sulfuric acid participates in the protonation of polypyrrole and provides sulfate counter-ions.

In the present synthesis, 20 mmol (1.34 g) of pyrrole was oxidised with 25 mmol (5.71 g) of ammonium peroxydisulfate in 200 mL of aqueous medium. The idealised stoichiometry (Figure 1) thus expects the 1.78 g yield of polypyrrole sulfate. Various amounts of nickel, 0.5–8 g, were introduced prior to the oxidant addition. The composite yields were close to each other in all three series (Table 1) carried out (1) in water, (2) in 0.1 M sulfuric acid, and (3) in water in the presence of methyl orange (MO). The compositions were practically independent of the reaction medium and well corresponded to the stoichiometric expectation (Table 2). Both components of the composite differ in density, 1.5 g cm^−3^ for polypyrrole [28] and 8.91 g cm^−3^ for nickel at 20 °C. One should keep in mind that electrical properties are controlled by volume fractions of components, and volume fractions of nickel are considerably lower than those based on weight (Table 2).

### 3.2. Morphology

**Figure 2:**
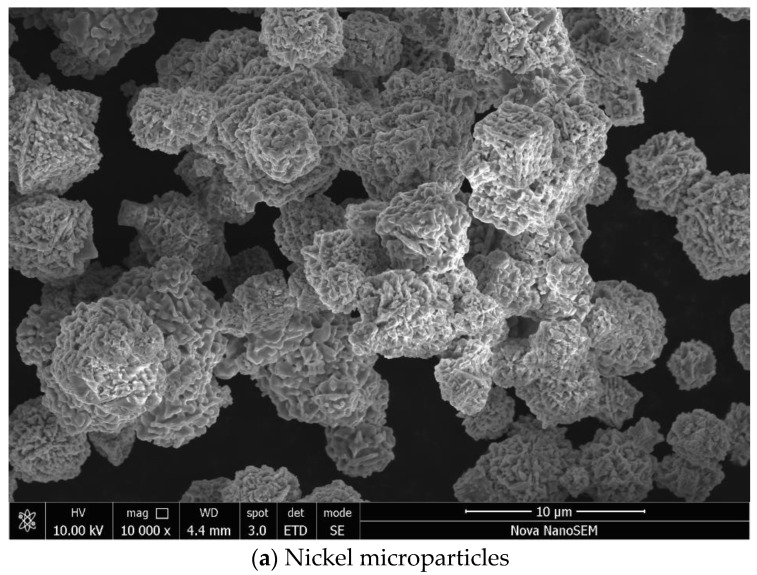

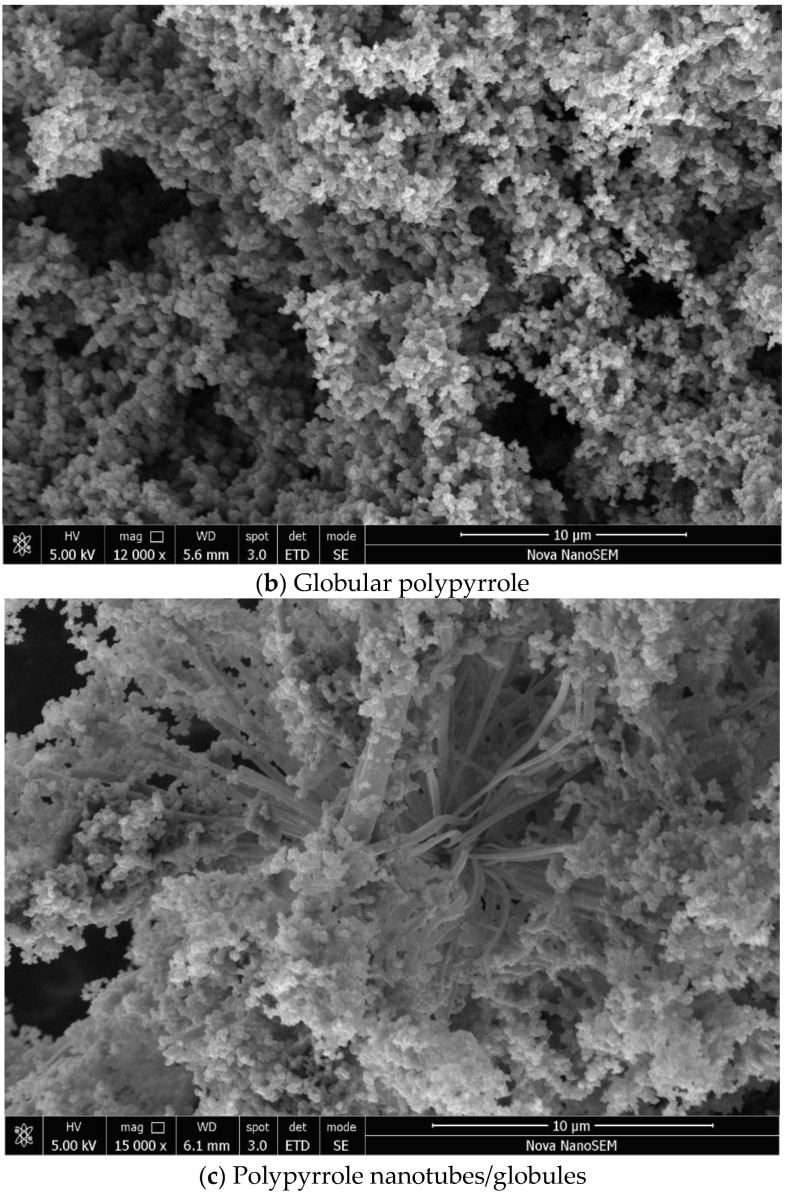
Scanning electron micrographs of composite components: (**a**) nickel and (**b**) globular polypyrrole, and (**c**) polypyrrole nanotubes/globules.

Nickel microparticles have a typical hedgehog morphology (Figure 2a) with the size of several micrometres and medium polydispersity. Polypyrrole was prepared in water without and with methyl orange, respectively. The former reveals the typical globular morphology with a particle size of 50–200 nm (Figure 2b); the latter resulted in the formation of polypyrrole nanotubes accompanied by a significant fraction of globular morphology (Figure 2c). It has to be stressed that uniform and highly conducting polypyrrole nanotubes are prepared with iron(III) chloride oxidant, and its replacement with ammonium peroxydisulfate in a present study led to definitely inferior morphology with only a limited occurrence of nanotubes.

**Figure 3:**
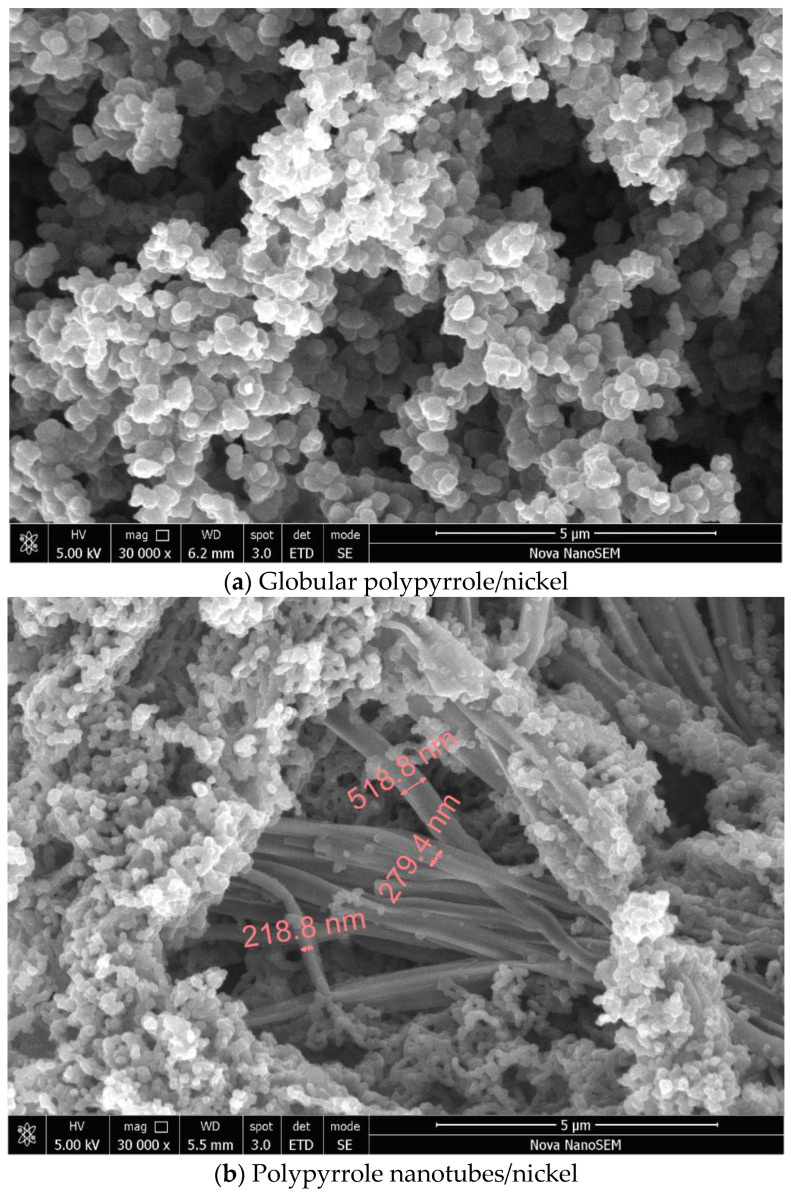
Scanning electron micrographs of composites composed of nickel (ca 50 wt%) and (**a**) polypyrrole globules or (**b**) polypyrrole nanotubes/globules.

The images of polypyrrole/nickel composites show only the presence of polypyrrole in globular form (Figure 3a) or its mixture with polypyrrole nanotubes with a diameter of about 100–500 nm and a length of 5–10 μm (Figure 3b). No nickel microparticles have been observed. During the polypyrrole synthesis, this polymer grows at the nickel surface, resulting in a complete coating of metal microparticles. As a result, nickel particles are dispersed in the polypyrrole matrix, and they stay apart from each other within the polymer phase. Such composite structure determines the electrical properties discussed below.

### 3.3. Spectroscopy

**Figure 4:**
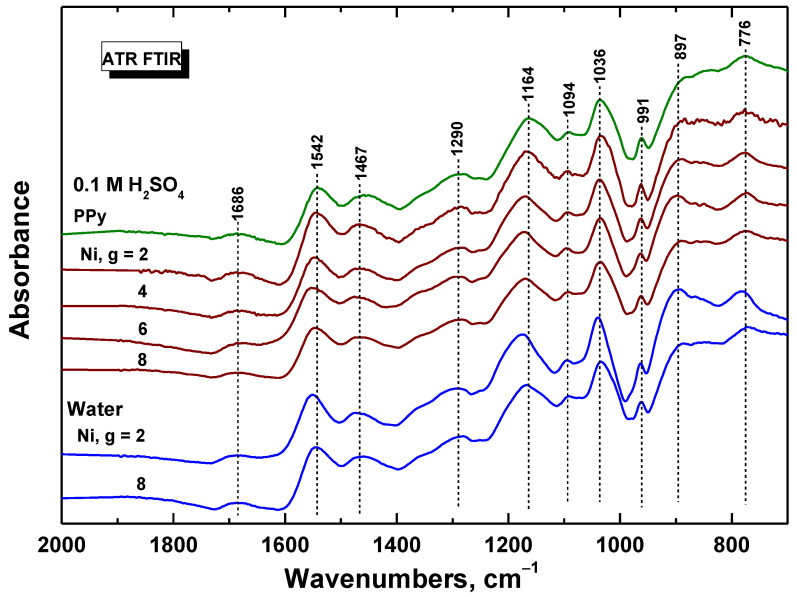
ATR FTIR spectra of polypyrrole and polypyrrole/nickel composites (2–8 g nickel) prepared in 0.1 M sulfuric acid or in water.

ATR FTIR spectrum of powdered polypyrrole prepared in the absence of nickel (PPy in Figure 4) corresponds to the protonated form of polypyrrole (Figure 1), which exhibits, in addition to a broad absorption band at wavenumbers above 2000 cm^−1^ (Figure 4), the main bands with local maxima situated at 1542 cm^−1^ (C–C stretching vibrations in the pyrrole ring), 1467 cm^−1^ (C–N stretching vibrations in the ring), 1290 cm^−1^ (C–H and C–N in-plane deformation modes), 1164 cm^−1^ (breathing vibrations of the pyrrole ring), 1094 cm^−1^ (breathing vibrations of pyrrole ring), 1036 cm^−1^ (C–H and C–N in-plane deformation vibrations), 991 cm^−1^ (C–H out-of-plane deformation vibrations of the ring), and at 897 cm^−1^ (C–C out-of-plane deformation vibrations of the ring). The maximum of the broadband detected at 1686 cm^−1^ was assigned to the presence of the carbonyl group previously attributed to the nucleophilic attack of water on the pyrrole ring during the preparation. The shape of polypyrrole spectra did not change with increasing amounts of nickel in the reaction mixture, i.e., nickel did not interfere with the formation of polypyrrole. A small shift of the maxima of the main bands to higher wavenumbers may indicate a slight deprotonation of the polypyrrole with an increasing amount of nickel. There is no observable difference between polypyrrole prepared in water and 0.1 M sulfuric acid (Figure 4). This is not surprising because sulfuric acid is generated as a by-product during the synthesis (Figure 1).

**Figure 5:**
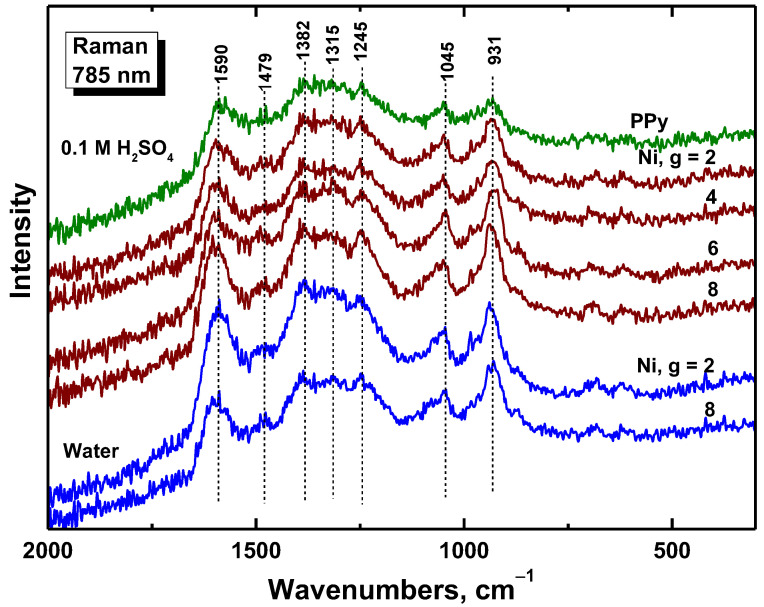
Raman spectra of polypyrrole (PPy) and polypyrrole/nickel composites (Ni, g = 2–8) prepared in water and in 0.1 M sulfuric acid.

Raman spectroscopy is the surface-dedicated method. In Raman spectra of polypyrrole prepared in the absence of nickel (PPy in Figure 5), we detect the bands of polypyrrole with local maxima at 1590 cm^−1^ (C=C stretching vibrations of polypyrrole backbone) and 1479 cm^−1^ (C–C and C=N stretching skeletal vibrations), two bands of ring-stretching vibrations at 1382 and 1315 cm^−1^, a band at 1245 cm^−1^ (antisymmetric C–H deformation vibrations), and a double-peak with local maxima at 1085 and 1045 cm^−1^ (C–H out-of-plane deformation vibrations, the second became sharper during deprotonation [29]). This is in agreement with the infrared spectra that suggest a slight deprotonation of the samples with an increasing amount of nickel.

### 3.4. Resistivity

The resistivity of various conducting powders is usually determined by the four-point method on compressed pellets. Free-standing pellets cannot be prepared for many materials, e.g., carbon black or carbon nanotubes, nickel or ferrite microparticles, and the characterisation has to be performed with powders under applied pressure. This is not the case of present composites, but the measurement of pressure dependences of resistivity provides additional information about the electrical properties of powders.

**Figure 6:**
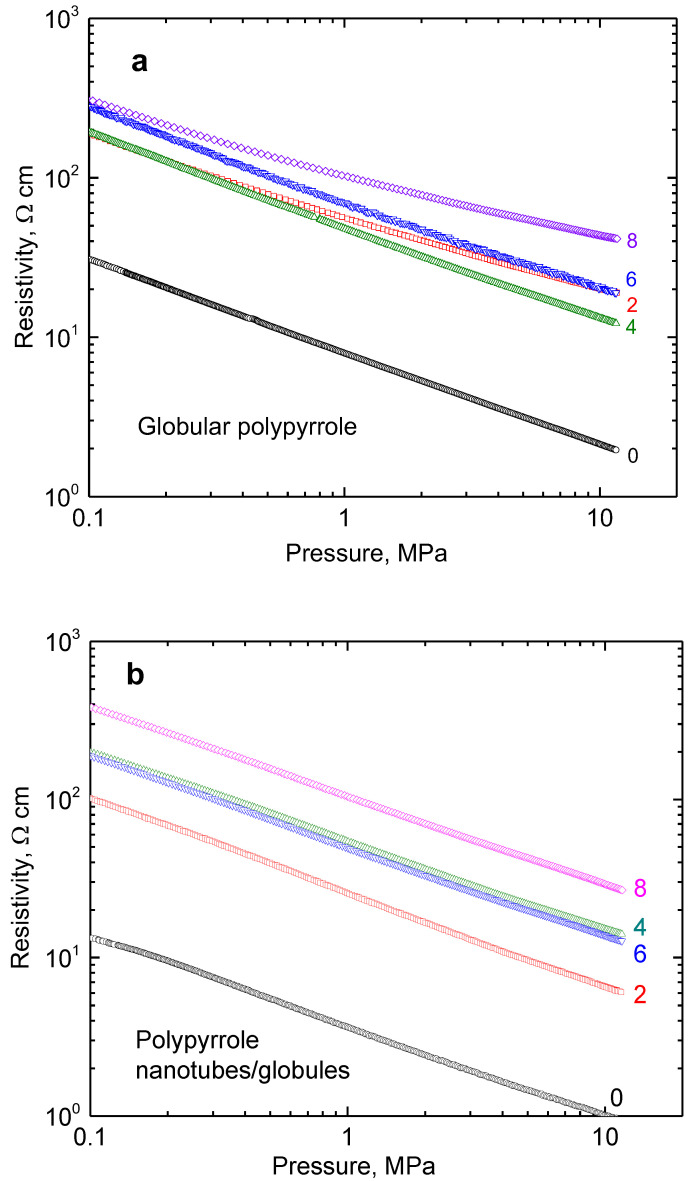

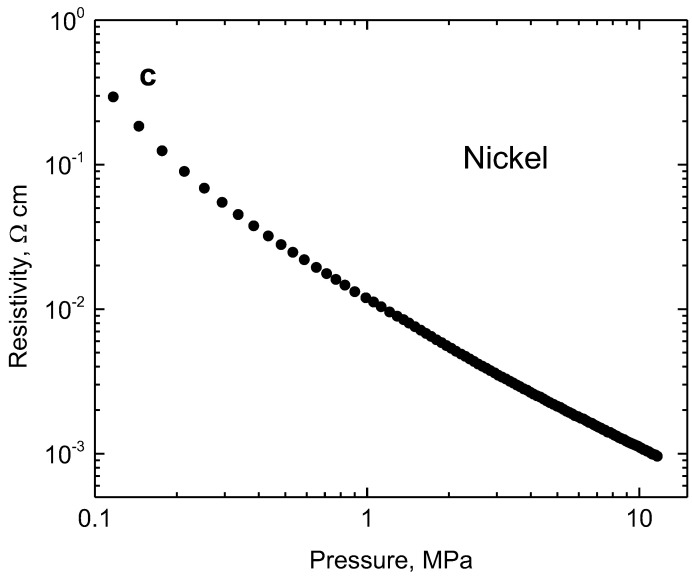
The pressure dependence of resistivity for (**a**) globular polypyrrole (0) and its polypyrrole/nickel composites (2–8 g nickel) and (**b**) for analogous polypyrrole nanotubes/globules composites with nickel. (**c**) The similar dependence for neat nickel microparticles.

Double-logarithmic dependences of resistivity on pressure are linear in the investigated range of 0.1–10 MPa for composites of both types of polypyrrole with nickel (Figure 6a,b). The resistivity of neat nickel particles recorded in the same manner is two orders of magnitude lower (Figure 6c). When compressed at 10 MPa, it was 1.43 × 10^−3^ Ω cm, which corresponds to 700 S cm^−1^ conductivity, in accordance with the earlier data [25]. The results then constitute an apparent paradox illustrating that the introduction of highly conducting nickel results in the *increase* of resistivity, and this becomes even more pronounced as the nickel content grows.

The explanation follows: Let us consider the classical case when the conducting metal particles are gradually introduced into a non-conducting polymer matrix. When their fraction is low, they are randomly distributed without any mutual contacts. In such a composite, no conducting pathways are present, and the composite would remain non-conducting despite the presence of dispersed conducting objects. When the content of metal particles becomes higher, their contacts start to occur, and finally, they generate the first conducting pathway at the so-called percolation threshold. The conductivity then starts to grow rapidly with increasing metal content.

The microstructure of the system under study, however, is different. Instead of neat metal particles distributed in the polymer matrix, the composite is constituted by metal particles *coated* with polypyrrole. This coating and any accompanying polypyrrole form the conducing matrix. This means that the coating prevents contact between metal particles; they will always be separated regardless of their fraction, and they cannot, therefore, create any conducting pathways and thus contribute to the overall composite conductivity. The conductivity of the composite is then determined by the conductivity of the polypyrrole matrix.

In addition, as the content of metal particles in the composite increases, the volume fraction of the matrix decreases accordingly, and consequently, the resistivity of the composite increases (Figure 6a,b). Such results produce an apparent paradox when the increasing fraction of conducting metal particles (but coated with polymer!) leads to an increase in the composite resistivity. A similar behaviour was observed earlier with nickel microparticles coated with polyaniline [25].

**Figure 7:**
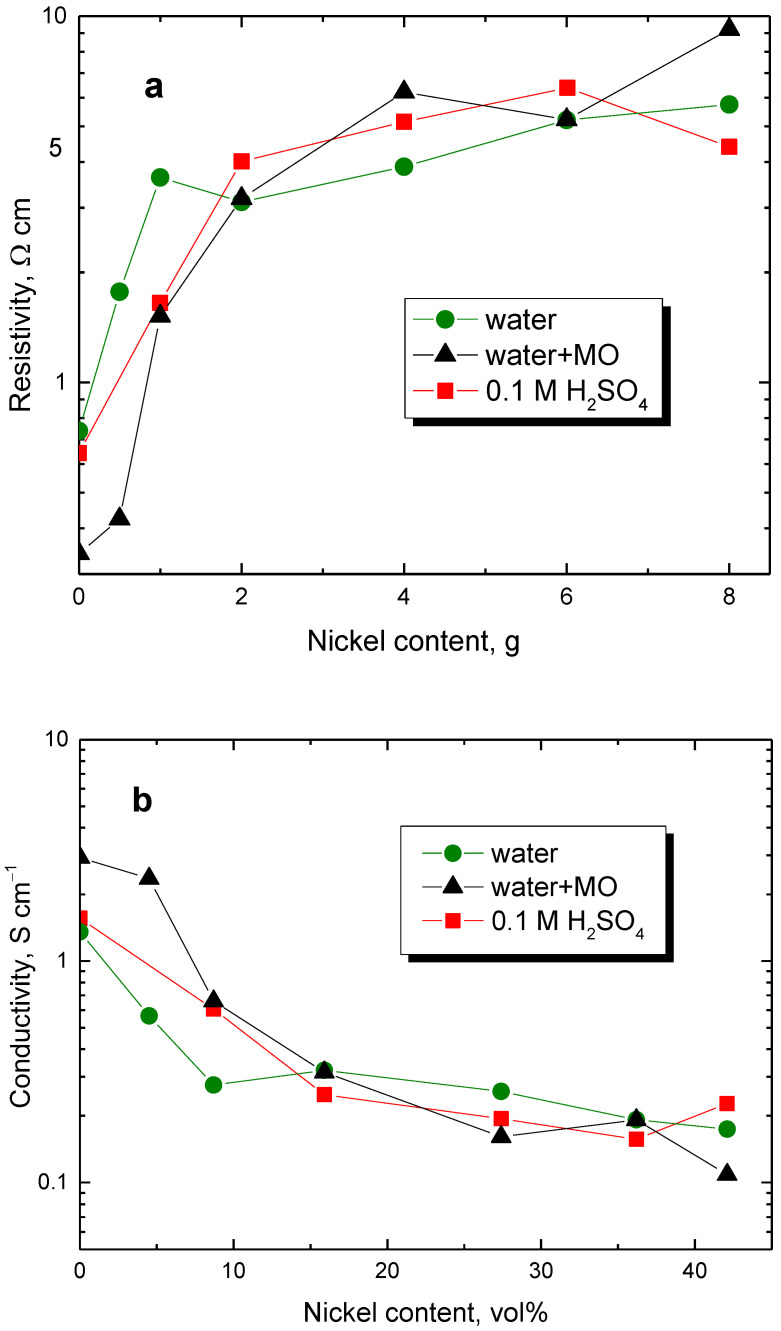
(**a**) Resistivity of polypyrrole/nickel composites determined in pellets in dependence on nickel content (in g) and (**b**) its reciprocal value, conductivity, on nickel content (in vol%). Preparation of globular polypyrrole in water (circles), nanotubes/globules in the presence of methyl orange (triangles), or globules in an acidic medium (squares).

In the present case, the standard compact pellets could also be prepared by compression at 527 MPa. The resistivity determined with them is several times lower than that found at 10 MPa pressure (Figure 7a). The resistivity of true polypyrrole nanotubes is lower compared with globular polypyrrole, but in the present study, the nanotubular morphology has not been fully developed. As far as the composites are concerned, the conductivity is, therefore, about the same within one order of magnitude regardless of the way of preparation and polypyrrole morphology.

Some readers may prefer plotting the conductivity against the volume fraction of nickel (Table 2, Figure 7b). The apparent paradox demonstrated then by the decrease in composite conductivity with increasing content of highly conducting nickel microparticles is obvious.

### 3.5. Mechanical Properties

**Figure 8:**
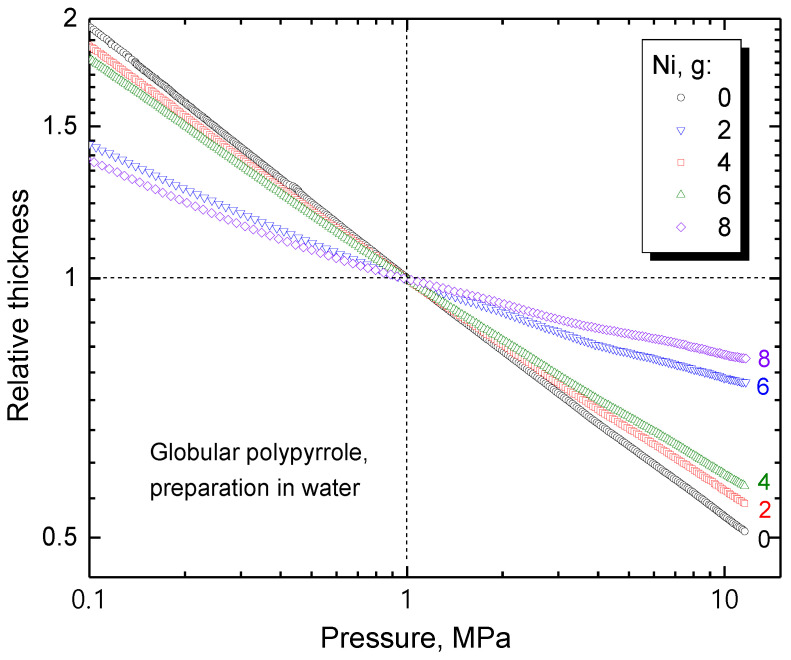
Pressure dependence of sample thickness relative to the thickness at 1 MPa for various contents of nickel.

The experimental setup used for the determination of resistivity also allows for the monitoring of sample thickness during compression (Figure 8). The pressure dependence of composites is close to linear in double-logarithmic presentation. The slope provides information about the fluffiness, i.e., the steeper it is, the easier the material is compressed. The decrease in the slope with increasing nickel content reflects the composite reinforcement with metallic microparticles.

### 3.6. Magnetic Properties

**Figure 9:**
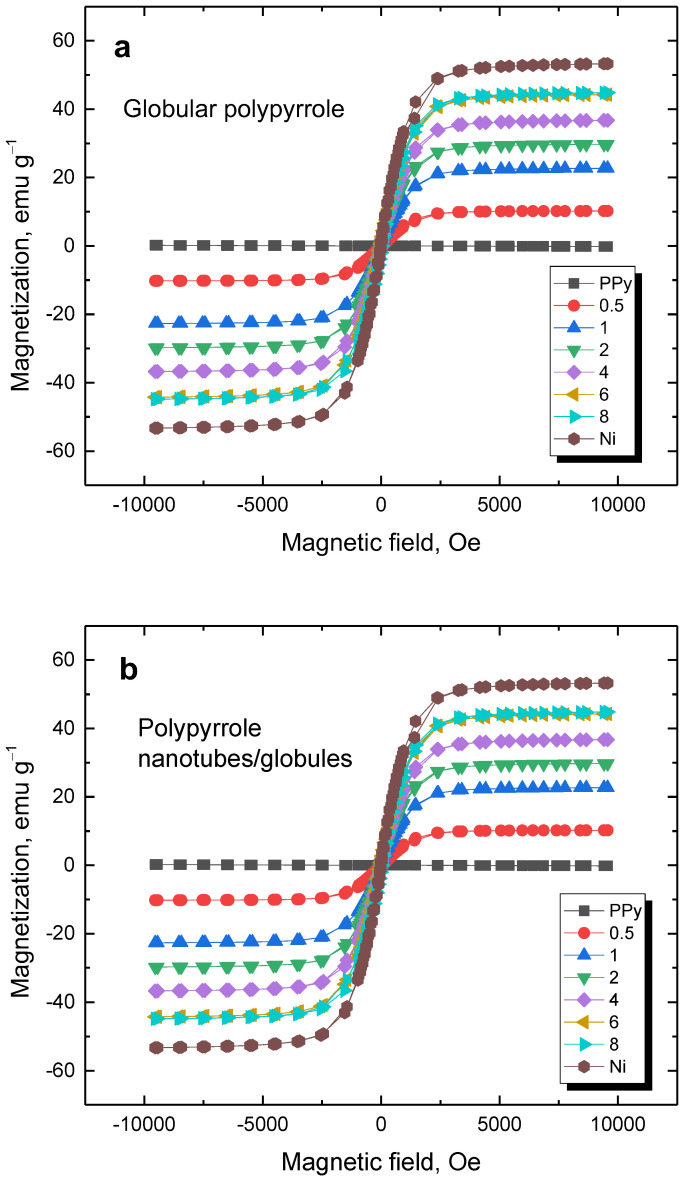
Magnetisation curves of (**a**) globular polypyrrole and (**b**) polypyrrole nanotubes/globules deposited on various amounts of nickel microparticles (in g).

The magnetisation curves reflect the properties of nickel (Figure 9). The saturation magnetisation increased with nickel content (Table 3). As expected, polypyrrole does not contribute to magnetic properties, and consequently, its morphology has no effect.

### 3.7. Magnetorheology

Magnetorheology deals with the liquid suspensions of magnetic particles and the changes in their rheology produced by applied magnetic fields. The prospective applications include magnetorheological dampers and mechanical shock absorbers in the automotive industry that become important with the expanding electromobility and availability of magnetic fields generated by electromagnets. Magnetorheological dampers are developed for military vehicles or aerospace. The composites are of promise in stimuli-responsive polymer systems in biomedical sciences [30], e.g., in human prostheses.

Due to the magnetic properties afforded by nickel, the composites with polypyrrole can be tested in the design of magnetorheological fluids composed of magnetic micrometre-sized particles and a non-magnetic liquid phase [31]. The viscosity of these suspensions increases by several orders of magnitude in less than a second when an external magnetic field is applied. Due to the density mismatch of the particles and the carrier medium, these systems suffer from sedimentation instability. The coating of metal particles with organics is one of the most common solutions [32]. It reduces the average density of composite particles and thus prevents the sedimentation of the solid phase [33,34]. The systems incorporating nickel have been reported seldom [35]. It has been observed that the magnetorheological effect depended on the morphology [36] and particle size [37] of the dispersed phase. The coating of nickel particles with polypyrrole reported in the present study may also be regarded as a way to alter the properties of the dispersed phase.

**Figure 10:**
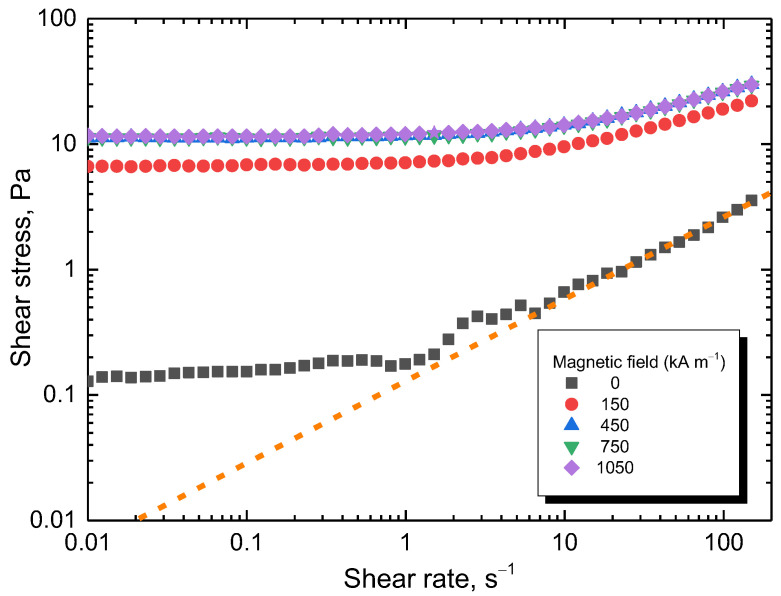
Flow curves at various magnetic fields for the composite prepared with 8 g of nickel. The dashed line represents the potential flow curve during the off-state if the shear band is not present.

The flow curves of the composite prepared with 8 g of nickel (=83.2 wt% Ni, Table 2) are shown in Figure 10. The shear stress increased with the intensity of the magnetic field up to 450 kA m^−1^. Then, the particles were magnetically saturated (as shown in the magnetisation curves), and the shear stress remained at approximately the same level. For the majority of the experimental window, the stress increased more than 10 times. A Bingham plastic behaviour was observed during the on-state. The particles form chain-like structures, granting the fluid a yield stress. However, above the shear rate of 1 s^−1^, the hydrodynamic forces started to compete with magnetostatic ones, eventually overtaking them. During the off-state below the shear rate of 10 s^−1^, flow instability was observed as a result of shear banding. This is a common behaviour for certain colloids especially when a plate–plate geometry is used. The selection of this geometry, however, is key for a uniform magnetic field. A dotted line has been drawn following the behaviour at high shear rates for comparison in case there was no shear banding.

**Figure 11:**
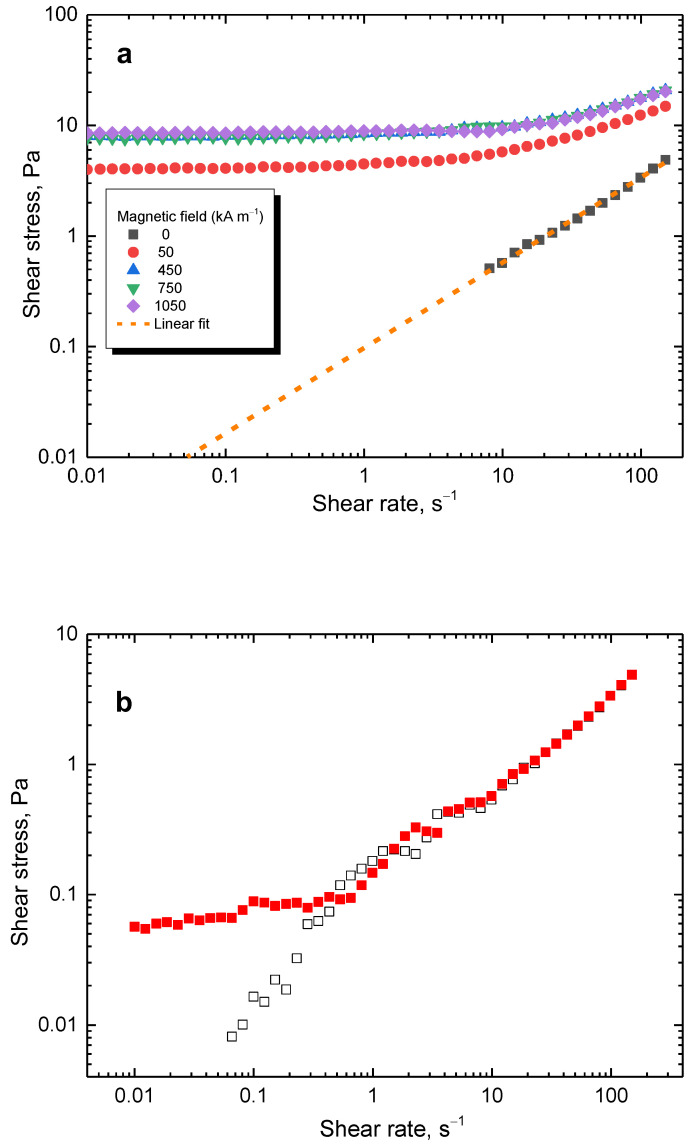
(**a**) Flow curves at various magnetic fields for the lowest 2 g nickel content. The dashed line represents a potential flow curve during the off-state if the shear band was not present. (**b**) Flow curves during the off-state. The full squares depict data with shear banding present, while open squares are those without shear banding.

The flow curves for the sample using the lowest 2 g content of nickel in the reaction mixture (=56.4 wt% Ni, Table 2) can be seen in Figure 11a. When a magnetic field was applied, the shear stress significantly increased, with the trend being similar to the sample shown in Figure 10. However, the shear stress increase is lower for the sample with low nickel content due to the lower content of the magnetic component. The same instabilities were observed (Figure 11b). However, for repetitions of the flow curves, a behaviour closer to reality is found, with the data not forming an unstable plateau. The data at lower shear rates are scattered due to the low torque measured by the instrument. Nevertheless, the trend is clear.

**Figure 12:**
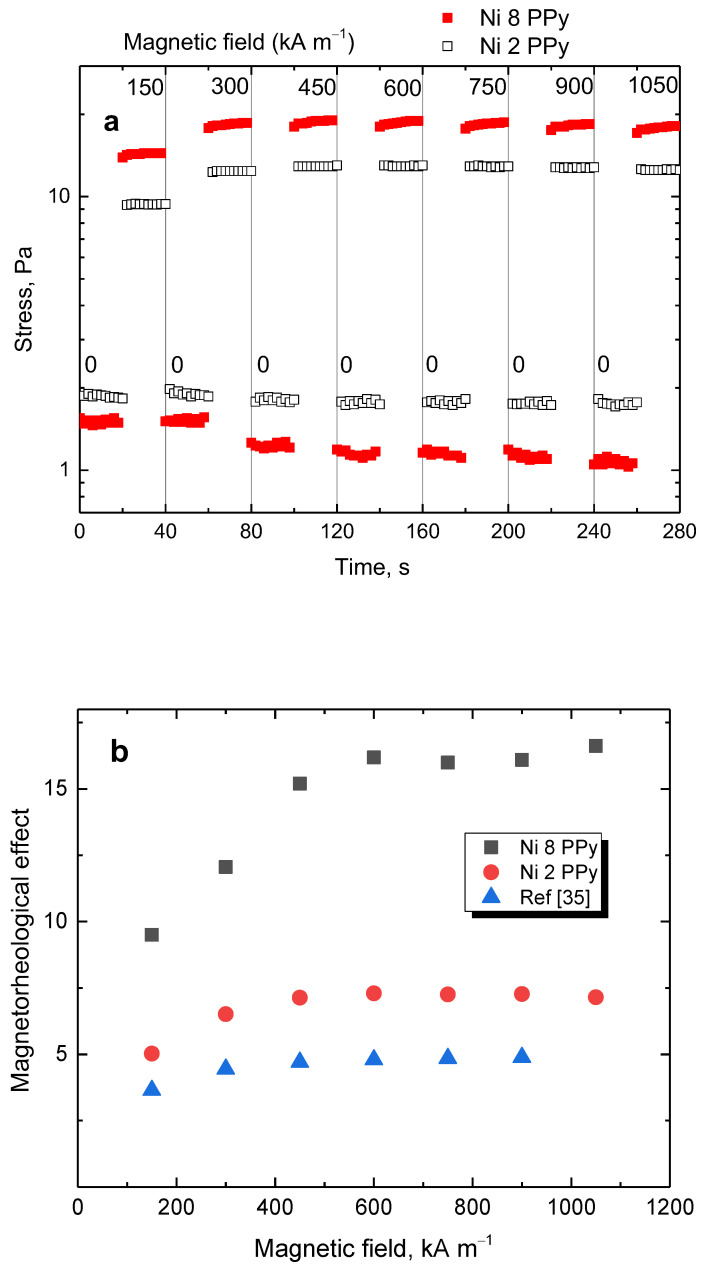
(**a**) Stepwise increase in the magnetic field under the shear rate of 50 s^−1^ for samples prepared with 8 g (full squares) and 2 g nickel (open squares). (**b**) The magnetorheological performance as a function of the magnetic field for composites prepared with 8 g (squares) or 2 g nickel (circles) and the comparison with data reported in the literature [35] (triangles).

Furthermore, both tested samples were exposed to a steady shear rate of 50 s^−1^ with the magnetic field turned on and off every 20 s and gradually increasing level (Figure 12a). The stress increased immediately after the magnetic field was applied. When the magnetic field was turned off, the stress reached its original values, showing great control. The sample with a high 8 g nickel content showed both lower off-state and higher on-state stress, promoting it as a better candidate for applications regarding magnetorheology. The average stress of the on- and off-states was calculated for both samples, and the magnetorheological performance was obtained by dividing the on-state stress by the off-state one (Figure 12b). The sample with 8 g nickel had approximately three times higher performance while, for both samples above 450 kA m^−1^, the performance remained the same as the nickel particles were magnetically saturated. It is important to note that there was a nickel ferrite-based magnetorheological fluid with a similar carrier [35]. Present composite particles have nearly two times higher performance with only 9 wt% loading, while the compared system [35] used 60 wt% particles.

## 4. Conclusions

The hybrid organic/inorganic composites displaying electrical conductivity and magnetic properties, polypyrrole-coated nickel microparticles, have been prepared and characterised. While the oxidation of pyrrole with iron(III) chloride in the presence of nickel does not provide desired composites due to the metal dissolution, the use of ammonium peroxydisulfate does. The resistivity of composite powders has been newly determined as a function of applied pressure. The conductivity measured on pellets was of the order of 10^−1^ S cm^−1^, i.e., slightly lower than that of neat polypyrrole. Electrical properties did not depend on the polypyrrole morphology, globules or nanotubes, or acidity of the reaction medium. The conductivity of nickel did not contribute to the overall conductivity of composites, which was determined only by the polypyrrole matrix. In fact, the conductivity moderately decreased with increasing content of well-conducting nickel. This apparent paradox is explained by the inability of nickel cores to produce conducting pathways, as they are always separated by polypyrrole coating. Magnetic properties were independent of polypyrrole content and morphology, and the magnetisation was proportional to the nickel content. The hybrid composites of this type may find application as competitive functional materials, e.g., in magnetorheology, as illustrated by present results.

## Figures and Tables

**Table 1:** The protocol for the oxidation of 0.1 M pyrrole with 0.125 M ammonium peroxydisulfate to polypyrrole in the presence of nickel microparticles at 20 °C.

Series	Medium	Additive	Morphology
1	water	–	globules
2	0.1 M H_2_SO_4_	–	globules
3	water	0.004 M MO	nanotubes/globules

**Table 2:** Composite composition expected from the reaction stoichiometry (Figure 1) in dependence on nickel mass entering 200 mL of the reaction mixture and the nickel content found in the composites prepared in water or 0.1 M sulfuric acid (globular polypyrroles), and in water in the presence of methyl orange (polypyrrole nanotubes/globules).

Nickel, g/200 mL	Expected Nickel Content		Nickel Content Found, wt%		
	wt% Ni	vol% Ni	Water	0.1 M H_2_SO_4_	Water + MO
0.5	21.9	4.5	18.3	–	22.9
1	36.0	8.7	35.0	–	33.6
2	52.9	15.9	56.4	52.8	53.1
4	69.2	27.4	70.9	70.2	70.7
6	77.1	36.2	78.5	77.7	78.3
8	81.2	42.1	83.2	84.7	83.4

**Table 3:** Coercivity, *H*_C_, remanent magnetisation, *M*_R_, and saturation magnetisation, *M*_S_, of polypyrrole composites prepared with various amounts of nickel, *Ni*, in water.

*Ni*, g	*H*_C_, Oe	*M*_R_, emu g^−1^	*M*_S_, emu g^−1^
Globular polypyrrole			
0.5	38.0	1.78	10.2
1	39.0	5.47	22.7
2	39.4	1.14	29.7
4	39.6	1.85	36.7
6	39.7	0.02	44.2
8	40.1	2.04	44.8
Polypyrrole nanotubes/globules			
0.5	38.9	2.26	11.7
1	38.5	4.87	15.3
2	38.8	7.19	17.6
4	39.9	1.48	28.7
6	40.3	2.00	39.3
8	40.2	3.82	44.3
Nickel			
Ni	41.7	0.2	53.3

## Data Availability

Data are contained within the article.
